# Update on the development of Group A Streptococcus vaccines

**DOI:** 10.1038/s41541-023-00730-x

**Published:** 2023-09-14

**Authors:** Sowmya Ajay Castro, Helge C. Dorfmueller

**Affiliations:** Division of Molecular Microbiology, School of Life Sciences, Dow Street, Dundee, DD1 5EH UK

**Keywords:** Protein vaccines, Conjugate vaccines

Group A Streptococcus (GAS) is a ubiquitous pathogen that causes a wide spectrum of human diseases. These include mild conditions such as tonsillitis and impetigo (with over 600 million cases *p.a*.), to scarlet fever and more severe invasive GAS diseases such as sepsis, toxic shock, and necrotising fasciitis. Invasive GAS (iGAS) affects more than 600,000 patients globally *p.a*., with a mortality rate of 25%^1^. Untreated or insufficiently treated, GAS infections can also trigger serious immune-related sequelae such as acute rheumatic fever, rheumatic heart disease (RHD), and inflammatory glomerulonephritis^2^. The IHME Global Burden of Disease estimates that globally there are more than 40 million cases of RHD^3^ with the majority of cases occurring in low- and middle- income countries (LMIC). Rheumatic fever is most common in children aged 5 to 15 years, and approximately 460,000 new cases occur each year. RHD contributes significantly to morbidity and mortality in LMIC, with over 300,000 deaths annually and over 10 million disability-adjusted life years^4^. Globally, GAS continues to be among the top ten infectious diseases responsible for causing mortality, highlighting the need to develop a GAS vaccine^5^. The WHO has taken the lead in this effort by publishing in 2018 a roadmap for the development of the first GAS vaccine^6^.

More than 250 GAS serotypes have been identified and annotated based on their *emm*-type genomic sequence that determines the serotype-specific M-protein (*emm*). Despite this serotype diversity, different geographical regions experience dissimilar *emm*-types. In the absence of a GAS vaccine, antibiotics are relied upon for the effective treatment and prevention of transmission. However, there are stark discrepancies in treatment and diagnosis between high-income countries (HIC) and LMIC, where vaccines would have an even greater impact. Although no cases of GAS resistance to beta-lactam antibiotics have been reported, there have been reports of reduced susceptibility^7,8^. Importantly, GAS laboratory surveillance has revealed no antibiotic resistance in the new clusters responsible for recent outbreaks and no newly-emerged *emm* types^9^. Over the past two decades, general antimicrobial resistance has increased, and certain GAS serotypes associated with severe infections have shown increased resistance to clindamycin and macrolides^10^. However, the excessive use of antibiotics for treating GAS pharyngitis and impetigo has played a major role in driving resistance in bystander pathogens^11^.

In 2019 a further positive movement towards the development and implementation of GAS vaccines was achieved through the establishment of two key initiatives: The Strep A Vaccine Global Consortium (SAVAC, https://savac.ivi.int/) and the Australian Strep A Vaccine Initiative (ASAVI, https:// www.asavi.org.au/). Their primary objective is to ensure the development of safe, effective, and affordable GAS vaccines, but they also facilitate connectivity among researchers, analysis of the value of a GAS vaccine, and development of business cases to encourage stakeholder involvement. Economic assessments in the United States estimated that the annual costs of iGAS and upper respiratory infections exceed $5 billion^12^. Therefore, implementing a vaccine programme in the HIC that reduces the GAS infection rate by 20% across all age groups could potentially save approximately $1 billion *p.a*. in the US alone, providing further incentive for funding bodies, including governments, to support GAS vaccine research.

As a result of the ASAVI and SAVAC initiatives, new collaborations have been formed, positive advocacy for GAS vaccines has increased, and funding commitments for GAS vaccine development have grown. Today, many different candidates are undergoing testing for safety and immunogenicity in rodents and rabbits, as outlined in review articles^13–15^. Notably, the first human challenge models are being developed^16^, which will allow for the evaluation of vaccine efficacy in human subjects. An important factor in GAS vaccine development is achieving cross-serotype protection, and the global GAS research community is actively exploring diverse pipelines of candidates and production methods to deliver on this challenge. GAS vaccine development approaches can be divided into two types: i) M-protein-based vaccine antigen and ii) non-M-protein-based vaccines targeting a diverse range of important GAS factors, including pyrogenic exotoxins such as *Streptococcus pyogenes* Adhesion and Division protein (SpyAD) and *Streptococcus pyogenes* cell envelope proteinase (SpyCEP), streptolysin O, C5a peptidase, and carbohydrate-based antigens^13–15^.

Three GAS vaccine candidates are recorded on http://www.globaldata.com/ as being tested in clinical trials or scheduled for Phase I clinical trials^14^. StreptAnova is a 30-valent vaccine candidate that targets M-proteins found on the surface of 30 GAS serotypes. Phase I clinical trials conducted in 2020 demonstrated the vaccine’s safety and ability to trigger an immunogenic response in healthy adults without causing autoimmunity. Individuals who received StreptAnova produced functional (opsonophagocytic) antibodies^13,17^. Another M-protein-based vaccine is StrepInCor, which consists of a 55-amino acid peptide from the C-terminal region of the M-protein. This region is highly conserved among GAS serotypes. Animal immunisation studies have shown high levels of specific antibodies with no cross-reactivity to cardiac proteins^18,19^. StrepInCor intends to start clinical trials by the end of 2023^14^. One promising approach presently in clinical trials is a combination of two synthetic peptides: two M-protein epitopes (the modified p*17 and J8) have been paired with an epitope from the streptococcal anti-neutrophil factor, Spy-CEP (K4S2)^20^. Preclinical models showed no cardiac or neurological pathologies using p*17- K4S2 and J8-K4S2, leading to the initiation of clinical trials in 2022^20,21^.

Several non-M-protein-based candidates are in pre-clinical trials to test the safety and immunogenicity in rodent models (see reviews in refs. ^14,15^). The recent success of mRNA-based COVID vaccines has stimulated exploration of this approach for GAS non-M-protein-based vaccine development. Researchers at the University of Queensland (Australia), in collaboration with Moderna, secured funding from the Leducq Foundation to develop an mRNA vaccine formulation building upon their protein-based multi-component vaccine candidates^22^.

In addition to protein- and peptide-based candidates, carbohydrate-based GAS vaccine candidates have been under development for the past decade (reviewed in ref. ^23^). This approach has been encouraged by the successful targeting of many *Streptococcus pneumoniae* serotypes through the development and deployment of capsule polysaccharide-based vaccines. However, the GAS capsule is mainly composed of hyaluronic acid, a molecule present in humans and therefore unsuitable for use as a human vaccine. Several research groups have targeted the GAS-specific surface carbohydrate, the Group A Carbohydrate (GAC). This could be considered a universal vaccine candidate as it is present in every GAS isolate and therefore is a promising candidate for targeting all GAS serotypes.

Considering the essential nature of its gene clusters and the complexity of remodelling carbohydrates for a pathogenic bacterium it is logical to test several GAC derivatives in several different systems^24–27^. Antibodies against GAC-derivatives protect against GAS infection in animal models of systemic and intranasal challenges^28^. These antibodies have also been shown to promote the clearance of multiple clinical strains of GAS in an opsonophagocytic killing assay^29^. Funding from CARB-X, Open Philanthropy and Wellcome supports researchers in developing and validating GAC-based vaccine candidates in animal models. CARB-X funding support has enabled Vaxcyte [24] and GSK Vaccines Institute for Global Health [26] to pursue combination vaccine approaches that target GAS specific proteins in conjunction with the GAC. This funding also encompasses Phase I clinical trials after reaching important milestones.

Recent outbreaks of iGAS in several countries have emphasised that our global goal must remain to develop an effective GAS vaccine. There are numerous potential benefits of a GAS vaccine, including prevention of infections, reduced incidence of complications, improved public health, and decreased use of antibiotics, thereby averting antimicrobial resistance.

Funding bodies, including global charities such as Wellcome, Leducq Foundation, Open Philanthropy, and CARB-X, as well as government funding from organisations in the USA, New Zealand, and Australia have significantly increased their financial commitments to rectify the long-standing neglect of GAS disease^30^. Such commitments are urgently needed to increase the likelihood of developing the first licensed GAS vaccine to stem this global problem within the next decade. Much work lies ahead, but a GAS vaccine is within reach and the mission is gaining the necessary momentum. Researchers worldwide, along with increased funding support, have made ground-breaking contributions to this field. The pivotal work led by SAVAC/ASAVI and their ambitious plans for the next five years, combined with the WHO published roadmap for a GAS vaccine providing a strategic framework for researchers, creates an ideal environment to make this mission a reality.
